# EGPA presenting as sudden cardiac arrest: a case report and review of cardiac manifestations

**DOI:** 10.3389/fimmu.2025.1749843

**Published:** 2026-01-15

**Authors:** Huihui Zhang, Miaolin Zhang, Tao Yu, Zhi Zhang, Mengyang Cai, Gang Wang, Weizong Zhang, Huamin Yu, Hong Yuan

**Affiliations:** 1 Department of Cardiovascular Medicine, First People’s Hospital of Linping District, Hangzhou, Zhejiang, China; 2 Department of Orthopedics, First People’s Hospital of Linping District, Hangzhou, Zhejiang, China; 3 School of Pharmaceutical Science, Zhejiang Chinese Medical University, Hangzhou, Zhejiang, China

**Keywords:** Adams-Stokes syndrome, eosinophilic granulomatosis with polyangiitis, eosinophils, mepolizumab, third-degree atrioventricular block

## Abstract

Eosinophilic granulomatosis with polyangiitis (EGPA) is a rare systemic vasculitis in which cardiac involvement is a primary cause of mortality. Complete heart block presenting as Adams-Stokes syndrome is a rare but critical complication. Notably, to our knowledge, EGPA initially manifesting as Adams-Stokes syndrome has not been previously documented, based on a comprehensive review of the literature. We report a 27-year-old female presenting with recurrent syncope and seizures. Laboratory tests revealed significant eosinophilia (49.5%), and cardiac workup confirmed third-degree atrioventricular block. A diagnosis of EGPA was established based on the 2022 ACR/EULAR criteria (score=13). Emergency treatment involved temporary pacing and methylprednisolone pulse therapy, followed by mepolizumab induction. Sinus rhythm recovered within 24 hours. During a two-month follow-up, the patient maintained remission with normalized eosinophil counts and improved cardiac function. This case highlights the importance of including EGPA in the differential diagnosis of unexplained high-grade heart block and supports the efficacy of early immunosuppressive therapy in reversing life-threatening cardiac complications.

## 1 Introduction

Eosinophilic granulomatosis with polyangiitis (EGPA) is a rare ANCA-associated vasculitis characterized by asthma, eosinophilia, and multi-organ involvement (1, 2). Cardiac involvement is the most serious complication and a leading cause of mortality (3).However, the initial manifestation as complete heart block (CHB) is exceedingly rare. A targeted literature review identified that complete heart block (CHB) or Adams-Stokes syndrome (ASS) has been reported in approximately 10–15 cases of EGPA, all occurring in patients with established disease or concurrent systemic features. To our knowledge, this is the first documented case in which recurrent ASS due to CHB served as the inaugural presentation of EGPA. This underscores the unique nature of the present case.

We report a 27-year-old female with EGPA who presented with recurrent Adams-Stokes syndrome due to complete (third-degree) atrioventricular block. This report aims to:1) highlight the importance of considering vasculitis in unexplained cardiac conduction abnormalities, particularly with eosinophilia; 2) demonstrate the application of the 2022 ACR/EULAR classification criteria; and 3) illustrate an successful management approach combining temporary pacing with targeted immunosuppression. The insights from this case are valuable for both cardiologists and rheumatologists encountering similar diagnostic and therapeutic challenges.

## 2 Case description

### 2.1 Clinical course

A 27-year-old female was admitted to the emergency department following “recurrent syncope accompanied by limb convulsions for 3 hours.” On physical examination, she presented with episodic loss of consciousness. Her vital signs were as follows: body temperature, 36.8°C; blood pressure, 96/62 mmHg. Auscultation of the lungs revealed bilateral moist rales. Cardiac examination showed a heart rate of 56 beats per minute with an irregular rhythm, and no pathological cardiac murmurs were detected. The abdomen was soft, without tenderness or rebound tenderness. There was no edema in the lower extremities, and muscle strength in all four limbs was normal.

Her past medical history was significant for chronic sinusitis for 2 years, which had been managed with intermittent therapy. She denied any history of smoking, alcohol consumption, drug or food allergies, or previous surgeries. Furthermore, there was no family history of premature cardiovascular disease or hereditary cardiac disorders.

The chronological sequence of key events is summarized in **Table 1**.

**Table 1:** Timeline of the patient’s clinical course.

Time point	Key event or Finding
Approximately 2 years prior to admission	Diagnosis of chronic sinusitis; onset of eosinophilia noted on laboratory tests.
6 months prior to admission	Diagnosis of “allergic sinusitis” at another institution; underwent sinus surgery. Received a short course of oral corticosteroids.
Between surgery and admission	Eosinophil count progressively increased after steroid discontinuation. No regular treatment.
Day of admission (0 hours)	Emergency presentation with recurrent syncope and convulsions.
On admission	ECG: Intermittent third-degree AV block. Emergency temporary pacing and IV methylprednisolone initiated.
Within 24 hours of admission	Return to sinus rhythm; pacemaker dependence ceased.
Hospital stay	Transition to oral glucocorticoids plus mepolizumab.
2-month follow-up	Normalized eosinophil count; glucocorticoids discontinued; maintained on mepolizumab.

### 2.2 Emergency laboratory and diagnostic investigations

Key laboratory findings from the emergency assessment are summarized in **Table 2**.

**Table 2:** Emergency laboratory findings on admission.

Test	Result	Reference range	Unit
Complete blood count			
White Blood Cell (WBC) Count	23.1	3.5-9.5	× 10^9^/L
Neutrophils	34	40.0-75.0	%
Lymphocytes	12.1	20.0-50.0	%
Eosinophils	49.5	0.4-8.0	%
Absolute Eosinophil Count	11.43	0.02-0.52	× 10^9^/L
Platelets	481	125 – 350	× 10^9^/L
Cardiac biomarkers			
Cardiac Troponin	1.788	< 0.04	ng/mL
B-type Natriuretic Peptide (BNP)	516.5	< 100	pg/mL
Key biochemistry			
Aspartate Aminotransferase (AST)	75.5	<35	U/L
Urea	2.7	2.8-7.6	mmol/L
Creatinine	65.9	49-90	μmol/L
Glucose	6.7	3.9-7.8	mmol/L
Creatine Kinase (CK)	538.8	26-140	U/L
Creatine Kinase-MB (CK-MB)	77.2	0-25	U/L
Lactate Dehydrogenase	463	120-250	U/L
(LDH)High-Sensitivity C-Reactive Protein (CRP)	54.4	0-8	mg/L

**Figure 1:**
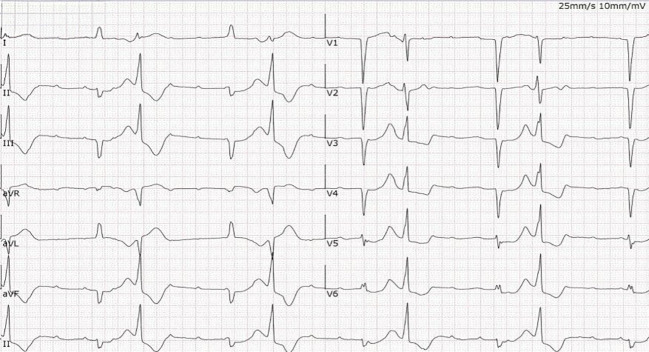
Admission electrocardiogram. The tracing shows sinus tachycardia with third-degree (complete) atrioventricular block and concomitant parasystole, corresponding to the clinical presentation of recurrent Adams-Stokes syndrome.

Electrocardiogram (ECG):The initial ECG showed sinus tachycardia with intermittent third-degree atrioventricular block (**Figure 1**).

**Figure 2:**
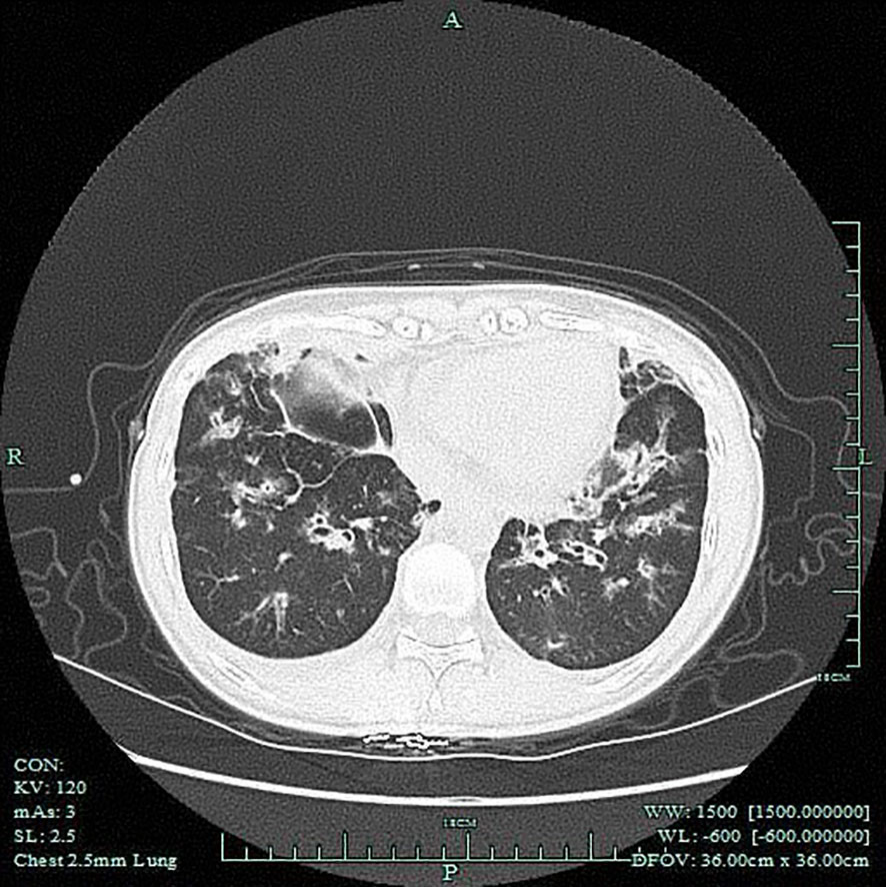
Chest CT scan on the day of admission. Images demonstrate bronchiectasis accompanied by extensive exudative opacities in both lungs, consistent with pulmonary involvement of EGPA.

Emergency Imaging Studies: Brain CT: No significant intracranial abnormalities were detected. An incidental note was made of increased density in the bilateral maxillary, ethmoid, and frontal sinuses, suggestive of sinusitis. Chest CT: Findings included bronchiectasis with associated infection, mild left-sided pleural thickening, and a small right-sided pleural effusion (**Figure 2**).

### 2.3 Emergency treatment

Following admission, the patient experienced recurrent episodes of Adams-Stokes syndrome, with cardiac monitoring revealing intermittent third-degree atrioventricular block. An emergency temporary pacemaker was subsequently implanted to stabilize the heart rate. Post-procedure, the patient had no further episodes of syncope and maintained stable vital signs.

### 2.4 Further diagnostic workup

To investigate the underlying etiology, the patient’s prior medical records from other hospitals were reviewed. Hematological tests from two years prior revealed eosinophilia. The patient had been diagnosed with allergic sinusitis at another institution and underwent sinus surgery six months ago. She received a short course of oral corticosteroid therapy; however, following its discontinuation, her eosinophil count progressively increased. No further regular treatment was pursued thereafter.

**Figure 3:**
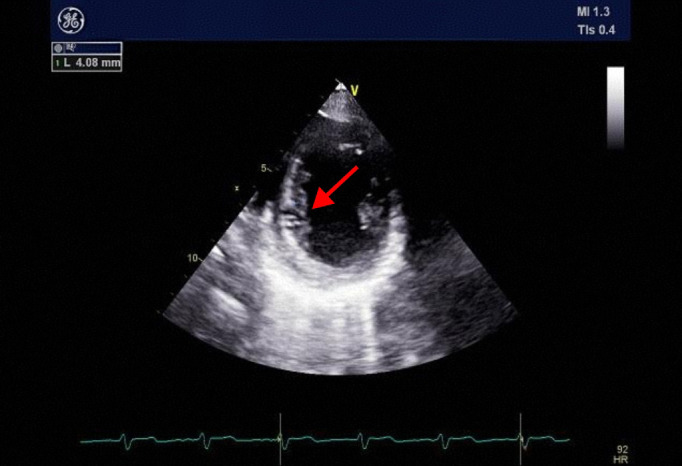
Transthoracic echocardiography on admission. The study reveals significant thickening of the left ventricular endocardium (arrow, max 4.08 mm), a characteristic finding suggestive of eosinophilic infiltration.

To elucidate the cause of eosinophilia, a detailed history and family history were obtained, which ruled out hereditary and other infectious causes. Serologic testing was negative for c-ANCA, p-ANCA, anti-proteinase 3 (PR3) antibodies, and a panel of parasitic antibodies. Transthoracic echocardiography revealed: 1. Mild thickening of the mid to apical segments of the left ventricular side of the interventricular septum(up to 4.08 mm) (**Figure 3**), and 2. Moderate mitral regurgitation.

**Figure 4:**
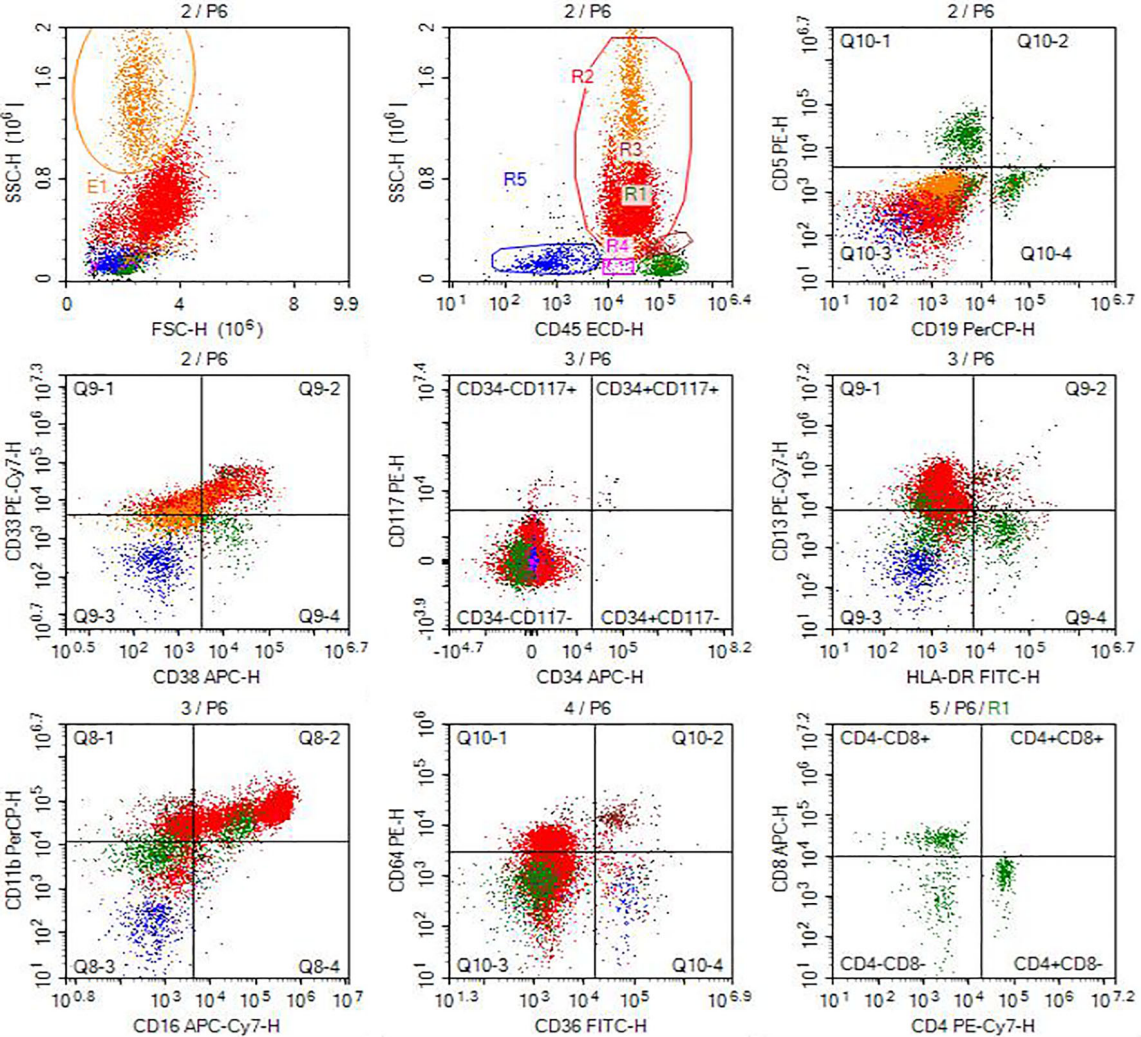
Immunophenotyping of the lymphohematopoietic system. Analysis shows an increased proportion of eosinophils (12.27% of nucleated cells) with normal granulocytic differentiation, supporting a reactive eosinophilia.

**Figure 5:**
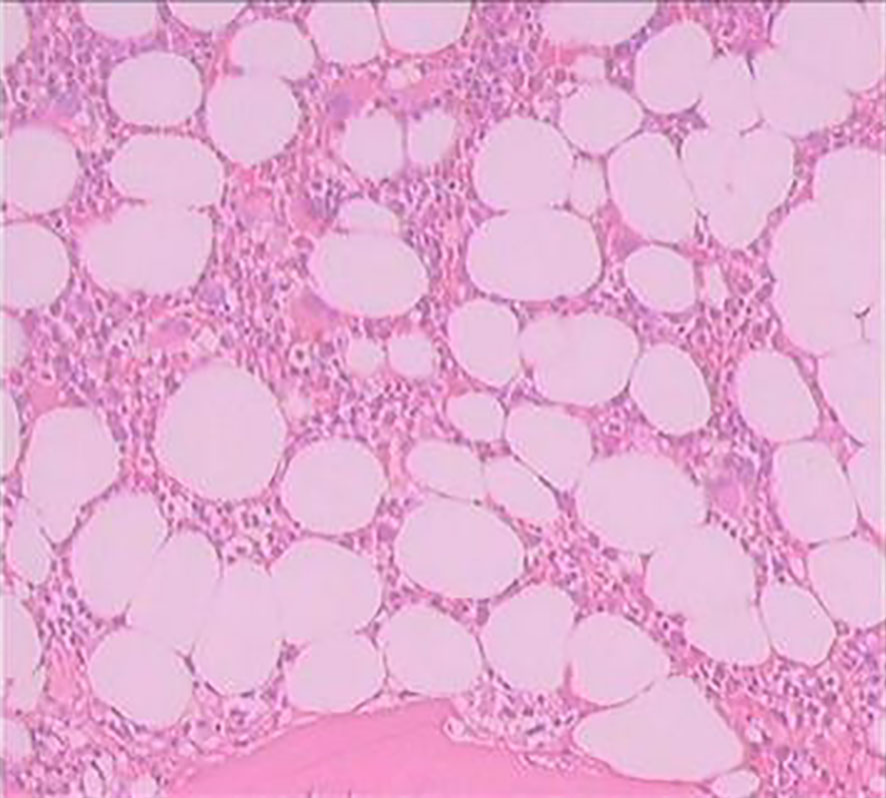
Bone marrow biopsy histopathology. The marrow is mildly hypocellular with preserved trilineage maturation and readily visible eosinophils, showing no evidence of a myeloproliferative neoplasm and supporting the diagnosis of reactive eosinophilia in EGPA.

To further assess for multi-organ infiltration by eosinophils, a bone marrow aspiration and biopsy were performed. Immunophenotyping of the hematopoietic system indicated an increased proportion of eosinophils (approximately 12.27% of nucleated cells) with normal granulocytic differentiation and no evidence of an aberrant blast population (**Figure 4**). Bone marrow biopsy showed mildly hypocellular marrow with trilineage maturation, no significant dysplasia, and readily observable eosinophils (**Figure 5**). Genetic testing revealed no mutations associated with myeloproliferative neoplasms, and no abnormalities were reported on tests for hematologic neoplasia-associated fusion genes, including BCR-ABL (P210, P190, and uncommon types).

**Figure 6:**
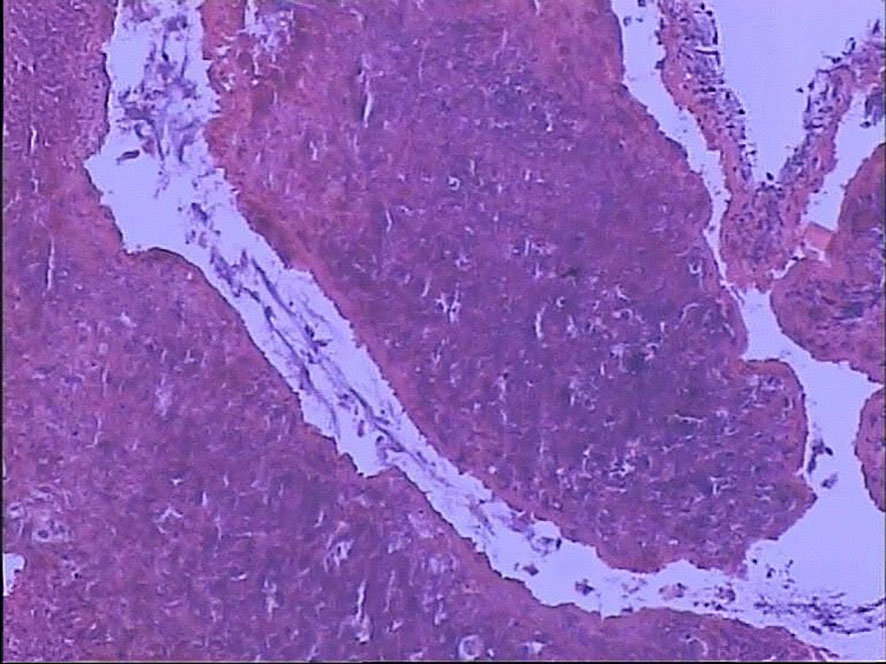
Bronchoscopic immunohistochemical staining. Stains for Periodic acid–Schiff (PAS) and acid-fast bacilli are negative, helping to exclude fungal and mycobacterial infections.

**Figure 7:**
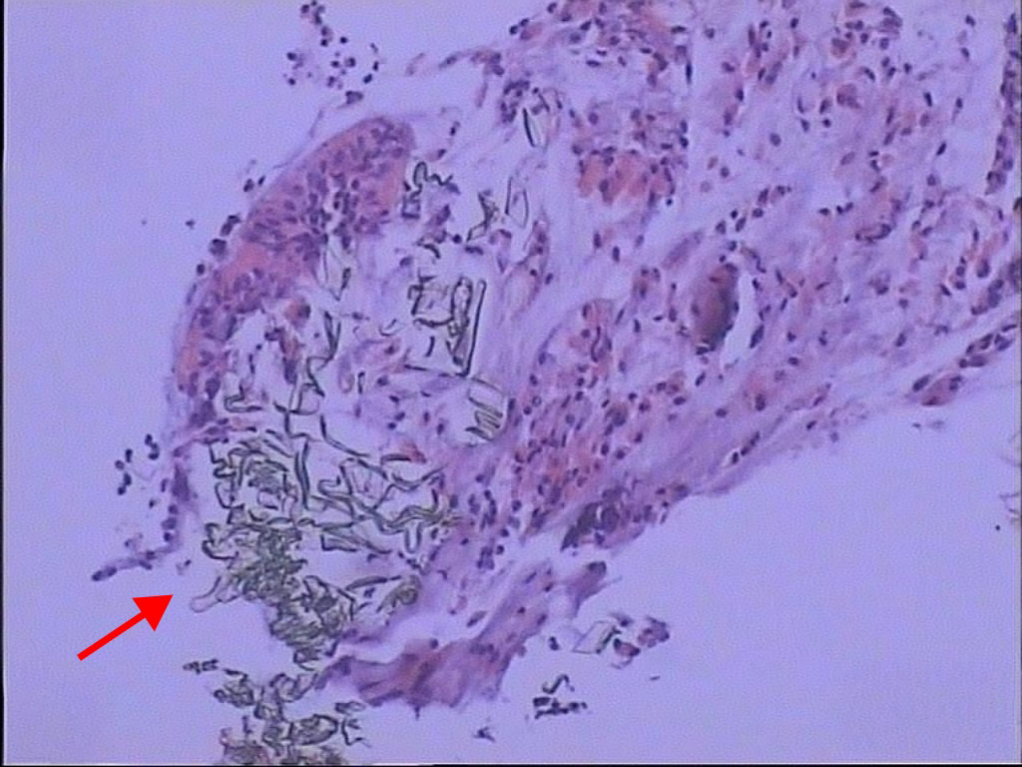
Bronchoscopic pathology (biopsy from the right lower lobe basal segment). Lung tissue shows fibroconnective tissue hyperplasia, chronic inflammation with multinucleated giant cell reaction, and scattered foreign body material(arrow). These findings are consistent with eosinophilic granulomatous inflammation and vasculitis, characteristic of EGPA pulmonary involvement.

Bronchoscopic immunohistochemical staining was negative for Periodic acid-Schiff (PAS) and acid-fast bacilli (**Figure 6**). Pathological examination of a biopsy from the right lower lobe basal segment revealed fibroconnective tissue hyperplasia projecting into alveolar spaces, accompanied by chronic inflammatory cell infiltration and multinucleated giant cell reactions. Scattered foreign body tissue and fragmented bronchial mucosal tissue were noted adjacent to the area, along with a small fragment of necrotic tissue (**Figure 7**). Pathogen metagenomic next-generation sequencing (PMseq-DNA) of the respiratory system detected no fungal, bacterial, mycobacterial, Mycoplasma/Chlamydia, parasitic, or viral pathogens.

### 2.5 Further management and follow-up

Based on the comprehensive investigations detailed above, which integrated the clinical presentation, laboratory findings, imaging characteristics, and histopathological evidence while excluding other differential diagnoses, the patient was diagnosed with eosinophilic granulomatosis with polyangiitis (EGPA). This diagnosis was established according to the 2022 American College of Rheumatology (ACR)/European Alliance of Associations for Rheumatology (EULAR) classification criteria (1). The patient’s cumulative weighted score was 13 points, definitively meeting the classification standard for EGPA.

The recurrent, life-threatening episodes of Adams-Stokes syndrome were attributed to cardiac involvement by eosinophilic infiltration, particularly affecting the endocardium of the interventricular septum, which resulted in third-degree atrioventricular block.

**Figure 8:**
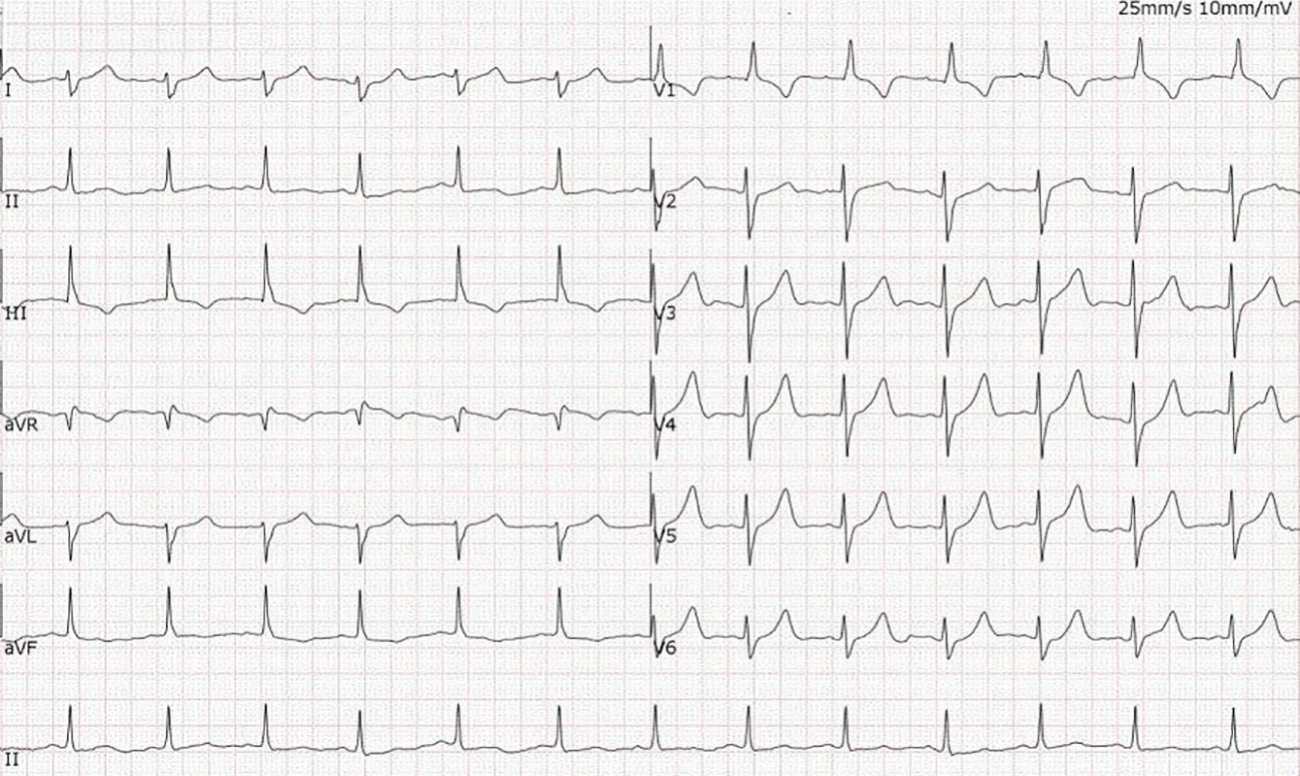
Follow-up electrocardiogram 24 hours after glucocorticoid therapy. The recording demonstrates restoration of sinus rhythm with incomplete right bundle branch block, indicating rapid improvement in cardiac conduction following immunosuppression.

Given the critical nature of her condition, management was initiated concurrently with temporary pacemaker implantation. This included intravenous methylprednisolone pulse therapy at a dose of 40 mg every 12 hours. Within 24 hours of initiating immunosuppressive therapy, continuous cardiac monitoring demonstrated a return to sinus rhythm with no further episodes of advanced heart block, and the patient no longer required dependence on the temporary cardiac pacemaker (**Figure 8**).

**Figure 9:**
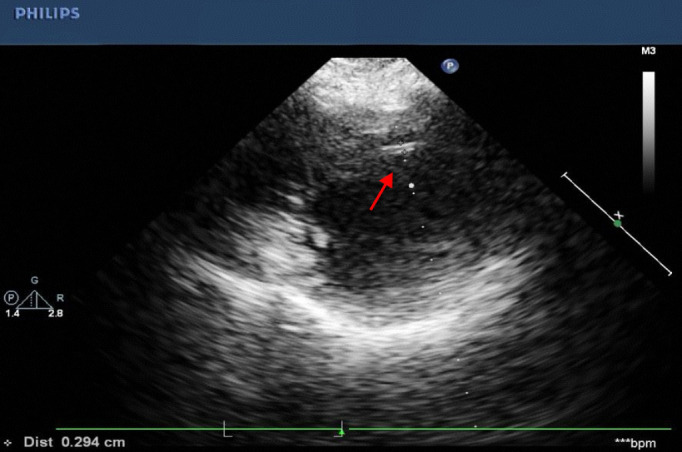
Follow-up echocardiogram at 2 months. The study shows reduction in the thickness of the septal endocardium to 2.94 mm (arrow), indicating response to treatment.

**Figure 10:**
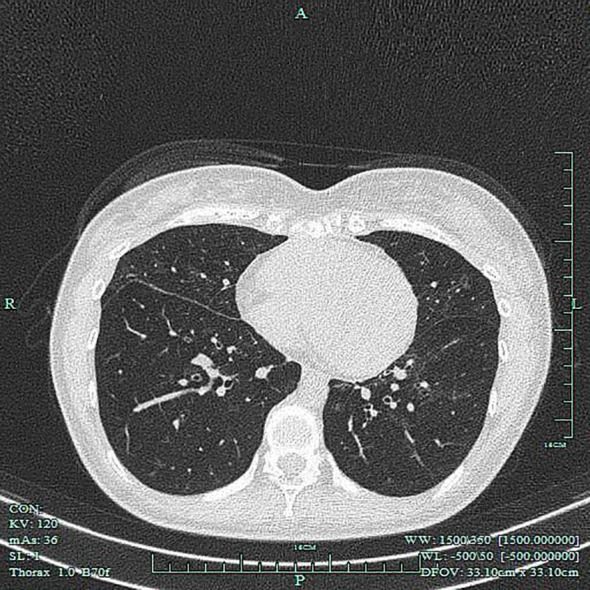
Follow-up chest CT scan at 2 months. The image reveals significant resolution of the previously noted pulmonary exudates, correlating with clinical improvement after therapy.

Following clinical stabilization, the treatment regimen was transitioned to a combination of oral glucocorticoids and mepolizumab. The glucocorticoid dose was subsequently tapered gradually and cautiously. At a two-month follow-up outpatient visit, her peripheral blood eosinophil count had normalized. A follow-up electrocardiogram showed sinus rhythm with complete right bundle branch block. Echocardiography revealed no left ventricular dilation or regional wall motion abnormalities, a normal left ventricular ejection fraction, and only mild residual thickening of the left ventricular septal endocardium (**Figure 9**). A repeat chest CT scan demonstrated significant improvement compared to the admission study (**Figure 10**), correlating with the patient’s reported resolution of cough and marked improvement in fatigue. Glucocorticoids have since been successfully discontinued, and the patient is currently maintained on mepolizumab 300 mg administered subcutaneously every 4 weeks. At the two-month follow-up, the patient reported a full return to her daily activities without any residual cardiac or respiratory symptoms, and her physical examination was unremarkable.

## 3 Discussion

We report a life-threatening presentation of eosinophilic granulomatosis with polyangiitis (EGPA), which initially manifested as Adams-Stokes syndrome due to third-degree atrioventricular block (AVB).This case highlights that high-grade AVB, though rare, can be the inaugural sign of severe cardiac EGPA and necessitates urgent immunosuppression alongside pacing support.

The underlying cardiac pathophysiology is a direct consequence of eosinophil-mediated tissue damage. This process is initiated by the pervasive infiltration of activated eosinophils into the myocardium and the cardiac conduction system (4). These eosinophils release a cascade of cytotoxic proteins, including major basic protein (MBP) and eosinophil cationic protein (ECP), along with pro-inflammatory cytokines, which collectively lead to myocyte necrosis, edema, and arteritis (5). The resultant inflammation and subsequent fibrosis can specifically disrupt the integrity of the conduction pathways, leading to electrical abnormalities (5–7).

Consequently, the most frequently observed cardiac manifestations of EGPA include pericarditis, myocarditis, and heart failure (8, 9).In contrast, high-grade atrioventricular block (AVB), particularly complete heart block requiring emergency pacing, is a rare but life-threatening presentation. Its exact prevalence is not fully established, but case series and literature reviews suggest that symptomatic conduction abnormalities are present in a minority of patients with cardiac involvement (3, 10). This is supported by a prospective study where conduction disturbances were a significant marker of poor outcome in cardiac EGPA (11, 12). The presence of AVB often signifies extensive inflammation or fibrosis in the interventricular septum, where the key components of the cardiac conduction pathway reside. Therefore, the onset of complete heart block should be regarded as a medical emergency and a red flag for severe, active cardiac EGPA, warranting immediate and aggressive immunosuppressive therapy alongside supportive pacing measures (3).

Establishing the diagnosis of EGPA in this context necessitated the systematic exclusion of alternative causes for eosinophilia and high-grade heart block. Key differentials that were actively considered and ruled out based on our investigations included: 1) hypereosinophilic syndromes of myeloproliferative origin, excluded by normal bone marrow cytogenetics and absence of blast cells; 2) parasitic or chronic infections, excluded by negative serology and metagenomic sequencing; and 3) other systemic vasculitides or granulomatous diseases, which were considered less probable given the patient’s negative c-ANCA/p-ANCA serology and the lung histopathology showing eosinophil-rich inflammation without typical features of those entities. This methodical exclusion process reinforces EGPA as the definitive underlying etiology.

In our patient case, consistent with this diagnosis, the marked peripheral eosinophilia, echocardiographic evidence of left ventricular endocardial thickening, and the exclusion of other hematologic disorders by bone marrow examination strongly support the diagnosis of eosinophilic infiltrative injury to the cardiac conduction system.

In cases of hemodynamically unstable high-grade AVB, temporary cardiac pacing is a life-saving cornerstone. Concurrently, high-dose corticosteroids should be initiated without delay to rapidly suppress vasculitic activity and eosinophil-mediated inflammation.

Following steroid pulse therapy, our patient was successfully transitioned to a maintenance regimen combining glucocorticoids with mepolizumab, ultimately allowing for steroid discontinuation. Mepolizumab, an anti-interleukin-5 monoclonal antibody, precisely targets the eosinophilic pathway. Clinical trials (13) have confirmed its efficacy in significantly increasing remission rates and reducing relapse risk in EGPA, making it an excellent agent for steroid-sparing or as an alternative to conventional immunosuppressants like cyclophosphamide. The critical importance of early and targeted intervention is further underscored by recent reports of severe eosinophilic cardiac involvement progressing to irreversible damage, highlighting the window of opportunity that exists with timely diagnosis and therapy (14).

**Strengths and Limitations:** The key strength of this report is its detailed documentation of a life-threatening, inaugural presentation of EGPA successfully reversed with emergency pacing and early targeted immunosuppression, providing a valuable management template. The main limitations are: 1) The lack of endomyocardial biopsy, which is often difficult to obtain and carries procedural risk, for direct histological confirmation of eosinophilic myocardial infiltration; 2) The absence of cardiac MRI due to the imperative for immediate life-saving intervention in an acutely unstable patient; and 3) as a single case report, the generalizability is inherently limited, underscoring the need for further collaborative studies. Despite these limitations, the diagnosis was robustly supported by congruent clinical, laboratory, and imaging findings.

**Clinical Implications:** Based on the life-threatening presentation and successful management of this case, we propose the following clinical implications:1) For Unexplained High-Grade Heart Block: An absolute eosinophil count should be urgently included in the initial laboratory workup. Significant eosinophilia (>1.5 × 10^9^/L) in this context is a red flag warranting immediate rheumatology consultation to evaluate for systemic vasculitis, particularly EGPA.2)For Acute Management: The concurrence of complete heart block and eosinophilia constitutes a medical emergency. Management must simultaneously address both issues: immediate temporary pacing for hemodynamic support and prompt initiation of high-dose corticosteroids to suppress vasculitic activity, without waiting for all diagnostic confirmations.3) For Long-term Therapy: Early introduction of targeted anti-IL-5 therapy (e.g., mepolizumab) should be considered in EGPA with severe cardiac involvement, as it facilitates steroid tapering, maintains remission, and may prevent progression to irreversible fibrotic damage.

## 4 Future perspectives

This case underscores a critical clinical imperative: Adams-Stokes syndrome as the primary manifestation of EGPA represents a rare but life-threatening emergency that demands heightened interdisciplinary vigilance. For cardiologists evaluating unexplained high-grade AV block, incorporating a simple eosinophil count into the initial workup could provide pivotal diagnostic clues warranting immediate rheumatology consultation. Conversely, rheumatologists should assert their role as key investigators in such complex presentations, driving the search for underlying systemic inflammation. Future collaborative studies through international registries are needed to establish optimized, evidence-based protocols for managing these severe EGPA phenotypes. Ultimately, fostering robust cardiology-rheumatology collaboration represents the most promising pathway toward timely diagnosis and improved survival in such extraordinary cases.

## 5 Conclusion

In conclusion, EGPA can present with life-threatening high-grade AVB. Early recognition, prompt pacing support, and rapid initiation of immunosuppressive therapy are paramount. This case illustrates the value of screening for systemic vasculitis in patients with cardiac conduction block and significant eosinophilia and demonstrates the favorable outcome of a mepolizumab-based targeted therapy strategy in inducing and maintaining disease remission.

## 6 Patient perspective

The patient, a young female, has achieved excellent recovery following critical presentation with Adams-Stokes syndrome. Her mother, who served as guardian during the acute phase, expresses profound gratitude for the life-saving intervention and clear communication throughout the treatment course. The family demonstrates full understanding of the EGPA diagnosis and commitment to the long-term management plan involving mepolizumab therapy.

## Data Availability

The datasets presented in this study can be found in online repositories. The names of the repository/repositories and accession number(s) can be found in the article/supplementary material.
